# Dysregulation of lncRNA TFAP2A‐AS1 is involved in the pathogenesis of pulpitis by the regulation of microRNA‐32‐5p

**DOI:** 10.1002/iid3.1312

**Published:** 2024-09-10

**Authors:** Mingming Liu, Weijing Jia, Lin Bai, Qiaolin Lin

**Affiliations:** ^1^ Department of Laboratory Center The First Hospital of Hebei Medical University Shijiazhuang China; ^2^ Department of Stomatology Shijiazhuang Fourth Hospital Shijiazhuang China

**Keywords:** inflammation, microRNA‐32‐5p, pulpitis, TFAP2A‐AS1, osteogenic differentiation

## Abstract

**Objective:**

This study was designed to evaluate TFAP2A‐AS1 expression in the dental pulp of teeth with or without pulpitis and to determine the function of TFAP2A‐AS1 in pulp cells.

**Methods:**

GSE92681 was analyzed to filter out differentially expressed lncRNAs. Pulp samples from teeth with pulpitis and healthy teeth (control) were examined using real‐time (RT) quantitative polymerase chain reaction (qPCR). Human dental pulp stem cells (hDPSCs) were cultured in a specific medium for osteogenic induction, or treated with lipopolysaccharide (LPS) to simulate inflammation. The viability and apoptosis of human DPSCs (hDPSCs) were determined by XTT assay and apoptosis detection kit. Inflammation was induced by LPS and assessed by measuring the expression and release of inflammatory cytokines after TFAP2A‐AS1 knockdown. Osteogenic differentiation of hDPSCs was investigated by determining expression levels of osteogenic markers and alkaline phosphatase (ALP) activity after TFAP2A‐AS1 overexpression. The downstream microRNA (miRNA) was predicted. Dual‐luciferase reporter was used to confirm the binding between miR‐32‐5p and TFAP2A‐AS1.

**Results:**

The expression of TFAP2A‐AS1 was evaluated in inflamed pulp using RT‐qPCR. TFAP2A‐AS1 had a discriminatory ability for healthy individuals and patients with pulpitis. The expression of TFAP2A‐AS1 decreased upon the osteogenic differentiation of hDPSCs, and increased upon the LPS induction. TFAP2A‐AS1 can reverse the osteogenic differentiation of hDPSCs, as evidenced by decreased levels of dentine sialophosphoprotein, dentin matrix protein−1, and ALP activity. TFAP2A‐AS1 knockdown can promote cell proliferation of hDPSCs and relieve LPS‐induced inflammation, as evidenced by decreased levels of TNF‐α, IL‐1β, and IL‐6. miR‐32‐5p was identified as a downstream miRNA of TFAP2A‐AS1.

**Conclusion:**

This study demonstrated the expression and potential function of TFAP2A‐AS1 in the human dental pulp. TFAP2A‐AS1 can inhibit odontogenic differentiation but promote inflammation in pulp cells.

## 1 INTRODUCTION

Teeth, although small, have a complex structure comprised of components like dental pulp, each with unique features and functions.^1^ Dental pulp is the soft tissue at the core of teeth, encased by the crown and root and is lined with a layer of odontoblasts.^2^ Teeth are prone to damage from intense mechanical and chemical stress, as well as dense microbiologic colonization. This damage usually occurs in the form of caries, periodontal disease and trauma.^3^ If the mineralized tissues of enamel and dentine are damaged, the dental pulp would becomes susceptible to microbial invasion.^4^ The toxins from bacteria can penetrate the dentinal tubules. The bacterial antigens and lipopolysaccharides (LPS) can cause immunological reactions and increase the levels of immunoglobulins, prostaglandins, and other pro‐inflammatory mediators in the infected pulp. In response to this insult, the dental pulp initiates a complex inflammatory response, leading to infection and inflammation. As bacteria in contact with the pulp, additional cell types of the pulp, such as stem cells, contribute great with a series of inflammatory‐defense mechanisms, which are critical for tissue homeostasis.^5^, ^6^ Stem cells exhibit both mesenchymal and neural characteristics.^7^ The pulpal stem cells may play a central role in the immunological responses of the dental pulp to oral microorganisms.^8^ A thorough understanding of molecular mechanism in pulpitis may lead to the identification of new therapeutic targets for this disease and enhance diagnostics strategies.

Epigenetics plays important roles in pulpitis, influencing aspects such as the inflammatory process and endodontic regeneration.^9^, ^10^ Epigenetic processes refer to mitotically and meiotically heritable changes, independent of the DNA sequence. These processes have a complex molecular basis that involves noncoding RNAs (ncRNAs).^11^ Since epigenetic modifications can be reversed, understanding their mechanisms could help identify new therapeutic targets for inflammatory diseases.^12^ Over recent decades, modulation of ncRNA, despite lacking protein‐coding function, has emerged as a new layer of gene regulation.^11^ ncRNAs can be classified as into two main types on length (shorter or longer than 200 nt)‐long noncoding RNA (lncRNA) and microRNA (miRNA).^13^ They play potential roles in dental pulp tissue, including odontogenic differentiation, immune response, and bone resorption.^14^, ^15^ For example, MEG3, an upregulated lncRNA in inflamed pulp and LPS‐treated human dental pulp cells, has been found to negatively affect inflammation and regeneration of the dentin‐pulp complex in pulpitis.^16^ It's worth noting that lncRNA can function as natural miRNA sponges, binding to miRNAs and competitively sequestering them from their target genes, a process referred to as competing endogenous RNAs (ceRNAs). For instance, upregulated lncRNA DUXAP8 is found in pulpitis and associated with miR‐18b‐5p sponging function, which exacerbates the pathogenetic condition.^17^

In this study, GSE92681 data set from GEO database was analyzed to screen the differentially expressed lncRNAs. Then, TFAP2A‐AS1 expression was evaluated in the healthy or inflamed dental pulp. Additionally, the function of TFAP2A‐AS1 in osteogenic differentiation and inflammation was examined.

## 2 MATERIALS AND METHODS

### 2.1 Collection of dental pulp tissue

This study was approved by the Institutional Review Boards of Shijiazhuang Fourth Hospital (approval no. 20230019). Each patient signed a written informed consent for use of the samples. Healthy dental pulp (*n* = 18) was obtained from wisdom teeth or premolars for orthodontic reasons. Simultaneously, inflamed dental pulp samples were extirpated from carious teeth with nerve broach in 37 patients with irreversible pulpitis (the American Association of Endodontists guidelines). The two groups were matched in age and gender (Table 1). The removed tissue was immediately placed in RNALater™ RNA Stabilization Reagent for Animal Tissue (Beyotime) and incubated at 4°C for 24 h. Then, the regent was discarded, and tissues were transferred to a prefrozen liquid nitrogen tube with a rotating cap. After rapid freezing with liquid nitrogen, the tissues were stored at −80°C.

**Table 1:** Clinical data comparison for subjects.

Items	Normal pulp (*n* = 18)	Inflamed pulp (*n* = 37)	*p* value
Age (year)	30.1 ± 10.8	33.2 ± 8.0	.29
Gender (female/male)	7/11	16/21	.76
Pain history (days)	—	7.8 ± 4.0	—

### 2.2 Cell culture, osteogenic induction, and LPS treatment

Human dental pulp stem cells (hDPSCs) from human sound third molars (Lonza) were cultured in Dulbecco's modified Eagle's medium (DMEM Sigma‐Aldrich) supplemented with 10% fetal bovine serum, 50 units/mL penicillin and 50 µg/mL streptomycin (Gibco), and maintained 37°C in a 5% CO_2_ incubator. Cells from passages 3 to 5 were used for experiments. To activate odontogenic differentiation, hDPSCs were cultured in a Human Dental Pulp Stem Cell Osteogenic Differentiation Medium (YaJi Biological) for 3, 7, and 14 days. mRNA levels of dentine sialophosphoprotein (DSPP) and dentin matrix protein (DMP)−1 were analyzed by real‐time (RT) quantitative polymerase chain reaction (qPCR), while alkaline phosphatase (ALP) was analyzed by ALP Diagnostics Kit (Yeasen). To simulate the inflamed microenvironment of dental pulp, hDPSCs were incubated with LPS (0.1, 1, 10 μg/mL) for 12 h. TNF‐α, IL‐1β, and IL‐6 were analyzed by RT‐qPCR and the responding ELISA kits (R&D Systems).

### 2.3 Cell transfection

Two siRNAs targeting TFAP2A‐AS1 (siTFAP2A‐AS1 and siTFAP2A‐AS1‐1) and siRNA negative control (siNC) were designed and synthesized by GenePharma, along with TFAP2A‐AS1‐overexpressed sequence (ovTFAP2A‐AS1) and negative control (ovNC). For transfection, hDPSCs were cultured in antibiotic‐free growth medium at about 60 confluence for 24 h and then transfected with 20 nM siRNA using Lipofectamine RNAiMAX (Invitrogen), followed by transfection efficiency verification based on the expression level of TFAP2A‐AS1 by RT‐qPCR.

### 2.4 RNA isolation and RT‑qPCR

The frozen healthy and inflamed dental pulp tissues were thawed and homogenized. Total RNA was isolated using RNAeasy™ Animal RNA Isolation Kit with Spin Column (Beyotime). Total RNA (400 ng) was subjected to reverse transcription, using the QuantiTect Reverse Transcription kit (Qiagen). The resulting cDNA was diluted 1:20 for RT‐qPCR using the SsoAdvanced Universal SYBR‐Green Supermix Kit (Bio‐Rad) and primers. After RT‐qPCR, quantification data were normalized using the geometric mean of the selected stable reference genes (Glyceraldehyde 3‐phosphate dehydrogenase for lncRNA and mRNA, and U6 for miR‐32‐5p), with the 2^−ΔΔCt^ method.

### 2.5 Cell proliferation and apoptosis assays

hDPSCs proliferation was determined by Cell Proliferation kit II (XTT) (Roche, Mannheim, Germany) abiding by the manufacturer's instructions. Cell apoptosis assays were completed by flow cytometry (CytoFLEX analyzer; Beckman Coulter), within 1 h of using FITC Annexin V Apoptosis Detection Kit (BD Biosciences) based on the recommendations from the manufacturer.

### 2.6 Bioinformatics analysis

The bioinformatics tool, lncRNASNP v2, was used to search the possible miRNA targets of TFAP2A‐AS1. Among the predicted miRNAs, miR‐32‐5p, known to be involved in osteogenic and adipogenic differentiation of dental pulp stem cells, was selected for further interaction studies with TFAP2A‐AS1.

### 2.7 Dual‐luciferase reporter assay

Based on the binding sites between TFAP2A‐AS1 and miR‐32‐5p, the wild‐type and mutant binding sequence regions of miR‐32‐5p on the fragment of TFAP2A‐AS1 were entrusted to Hanbio for chemical synthesis and inserted into pmirGLO luciferase reporter vectors (LMAI Bio). hDPSCs were transfected with reporter plasmids (wild or mutant TFAP2A‐AS1) and miR‐32‐5p mimics or mimic negative control (NC). After 24 h of transfection, the cells processed as per the instructions of Dual‐Luciferase Reporter gene assay kit (Promega). The activities of renilla luciferase and firefly luciferase were then measured using a GloMax luminometer (Promega).

### 2.8 Statistics

All data were transferred to statistics software (GraphPad Prism 9) for analysis, and the Kolmogorov‐Smirnov normality test was applied. Data that were not normally distributed required Mann‐Whitney method for parametric analysis. Normally distributed data were compared using either one way ANOVA or *t* tests. The correlation analysis between TFAP2A‐AS1 and miR‐32‐5p was analyzed using Pearson's correlation coefficient. The predictive performance of TFAP2A‐AS1 was assessed by receiver operating characteristic (ROC) curve analysis, represented as the area under the curve (AUC). When *p* < .05, the difference was statistically significant.

## 3 RESULTS

### 3.1 TFAP2A‐AS1 expression in healthy and inflamed dental pulp

**Figure 1:**
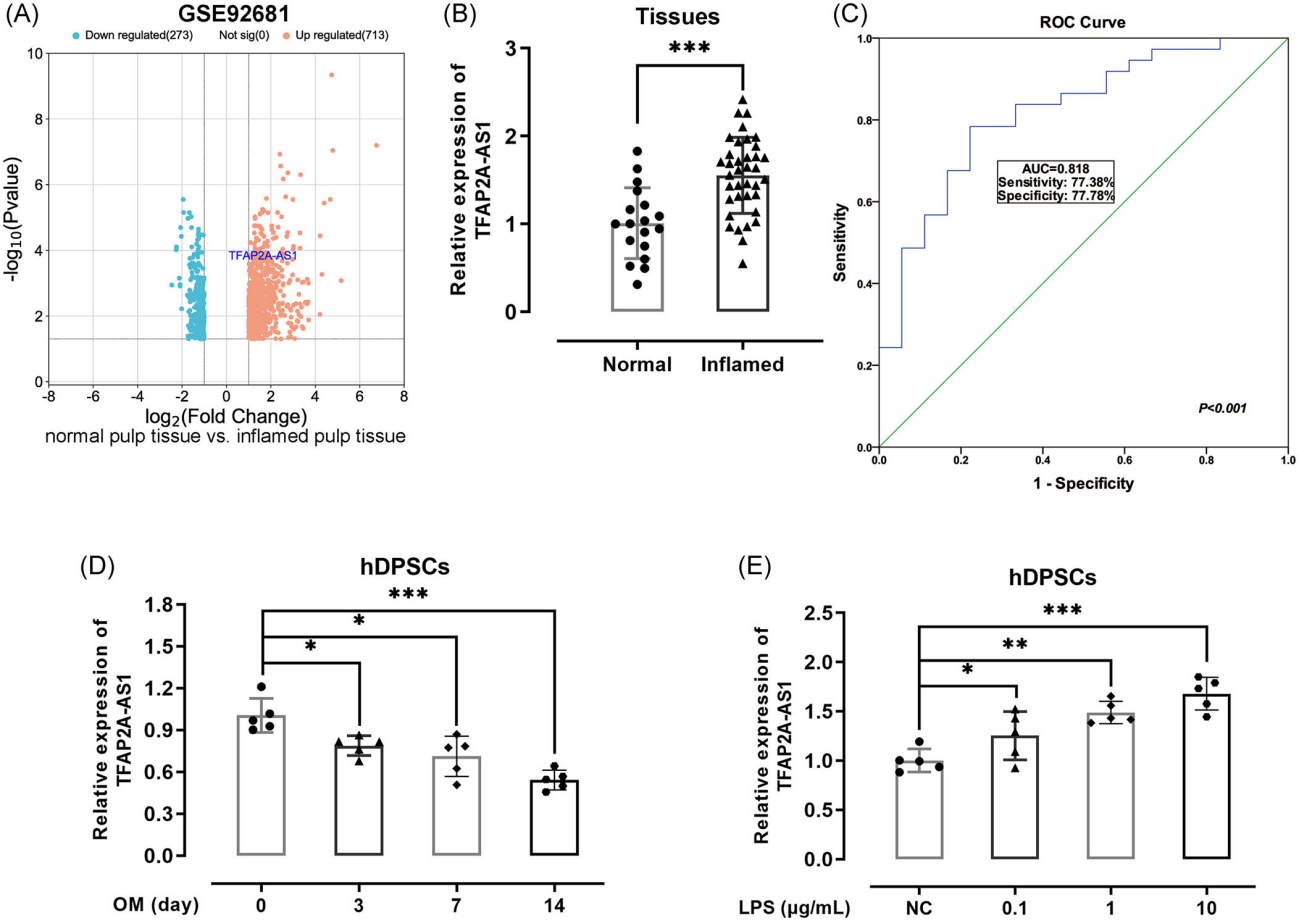
The expression levels of TFAP2A‐AS1 in dental pulp. (A) TFAP2A‐AS1 was one of the differentially lncRNA in GSE92681 data set. (B) TFAP2A‐AS1 expression level in healthy and inflamed pulp tissue was determined by real‐time quantitative polymerase chain reaction (RT‐qPCR). (C) The area under the curve (AUC) was analyzed by receiver operating characteristics (ROC) in predicting pulpitis. (D) TFAP2A‐AS1 expression in osteogenic‐differentiating hDPSCs was assayed by RT‐qPCR assay. (E) The expression of TFAP2A‐AS1 in LPS‐induced hDPSCs was determined by RT‐qPCR. *p < .05, ***p < .001. hDPSCs, human dental pulp stem cells. LPS, lipopolysaccharide.

TFAP2A‐AS1 was identified as a differentially expressed lncRNA in GSE92681 (Figure 1A). Then, the expression level of TFAP2A‐AS1 was assessed in the healthy and inflamed dental pulp. Results showed that pulpitis caused a sharp significant (*p*  <  .001) elevation in TFAP2A‐AS1 expression level in patients with inflamed dental pulp when compared with the healthy ones (Figure 1B). The TFAP2A‐AS1 level manifested as a well‐distinguishing tool for pulpitis and health, with an AUC of 0.818 (Figure 1C). During osteogenesis of hDPSCs, the TFAP2A‐AS1 level decreased (*p*  <  0.05) upon the culture time (Figure 1D). LPS exposure dose‐dependently increased the expression of TFAP2A‐AS1 in hDPSCs (Figure 1E).

### 3.2 TFAP2A‐AS1 overexpression inhibited osteogenesis in hDPSCs

**Figure 2:**
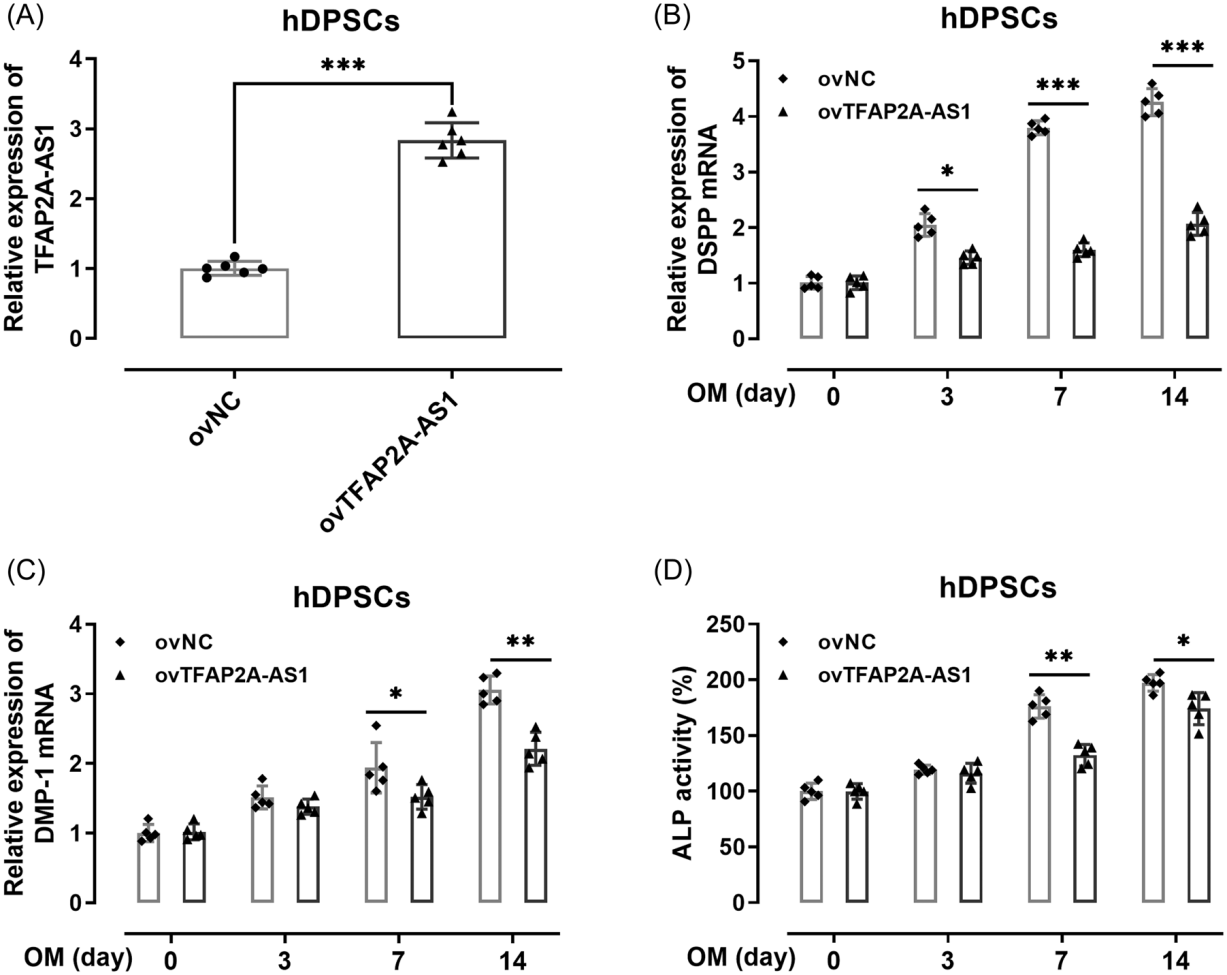
Overexpression of TFAP2A‐AS1 impeded odontogenic differentiation of hDPSCs. (A) TFAP2A‐AS1 expression level increased upon the transfection by RT‐qPCR. The mRNA expression of DSPP (B) and DMP‐1 (C) was analyzed by real‐time quantitative polymerase chain reaction (RT‐qPCR). (D) Alkaline phosphatase (ALP) activity assay was determined by ALP Diagnostics Kit. *p < .05, ***p < .001. hDPSCs, human dental pulp stem cells.

The influence of TFAP2A‐AS1 overexpression on the osteogenesis of hDPSCs was evaluated by the expression of osteogenic markers, including DSPP mRNA, DMP‐1 mRNA, and ALP activity. TFAP2A‐AS1 was overexpressed successfully (Figure 2A). According to the results, the seeded cells without TFAP2A‐AS1 overexpression had higher DSPP mRNA (*p*  <  .05) and DMP‐1 mRNA (*p*  <  .05) expression levels (Figure 2B,C). Analysis of ALP activity also showed a significant decrease (*p*  <  .05) in ALP activity due to TFAP2A‐AS1 overexpression (Figure 2D). These results demonstrated that TFAP2A‐AS1overexpression of in hDPSCs can inhibit hDPSCs differentiation into osteoblasts.

### 3.3 TFAP2A‐AS1 knockdown reversed LPS‐induced effects in hDPSCs

**Figure 3:**
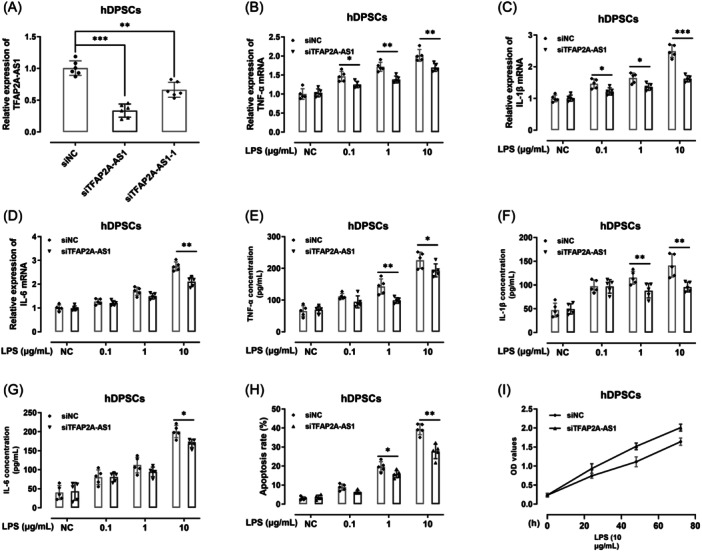
Knockdown of TFAP2A‐AS1 prohibited the inflammasome and cell apoptosis, but promote cell proliferation of hDPSCs. (A) TFAP2A‐AS1 expression level decreased upon the transfection of siRNA by RT‐qPCR. Expression of TNF‐α (B), IL‐1β (C), and IL‐6 mRNA (D) in hDPSCs was analyzed by real‐time quantitative polymerase chain reaction (RT‐qPCR). TNF‐α (E), IL‐1β (F), and IL‐6 (G) levels in culture supernatants of hDPSCs were determined by responding ELISA kits. (H) Cell death assay of transfected or non‐transfected hDPSCs. (I) Cell proliferation of hDPSCs, transfected or non‐transfected, was assayed by Cell Proliferation kit II (XTT). *p < .05, **p < .01, ***p < .001. hDPSCs, human dental pulp stem cells.

The impact of TFAP2A‐AS1 knockdown on LPS‐induced effects in hDPSCs was evaluated through the levels of inflammatory factors (TNF‐α, IL‐1β, and IL‐6), cell proliferation, and cell apoptosis. In comparison with siNC group, the expression level of TFAP2A‐AS1 was sharply decreased in siTFAP2A‐AS1 group (*p*  <  .001) and siTFAP2A‐AS1‐1 group (*p*  <  .01) (Figure 3A). Compared to siNC group, downregulation of TFAP2A‐AS1 in hDPSCs greatly reduced the mRNA expression levels of TNF‐α (Figure 3B), IL‐1β (Figure 3C), and IL‐6 (Figure 3D) (*p*  <  .05), as well as their protein levels (*p*  <  .05) (Figure 3E,F,G). TFAP2A‐AS1 knockdown significantly decreased (*P*  <  0.05) hDPSCs cell apoptosis (Figure 3H), while hDPSCs proliferation increased upon TFAP2A‐AS1 downregulation (Figure 3I). Consequently, downregulation of TFAP2A‐AS1 can protect hDPSCs from LPS‐induced inflammation and injury.

### 3.4 miR‑32‐5p was a direct target of TFAP2A‐AS1

**Figure 4:**
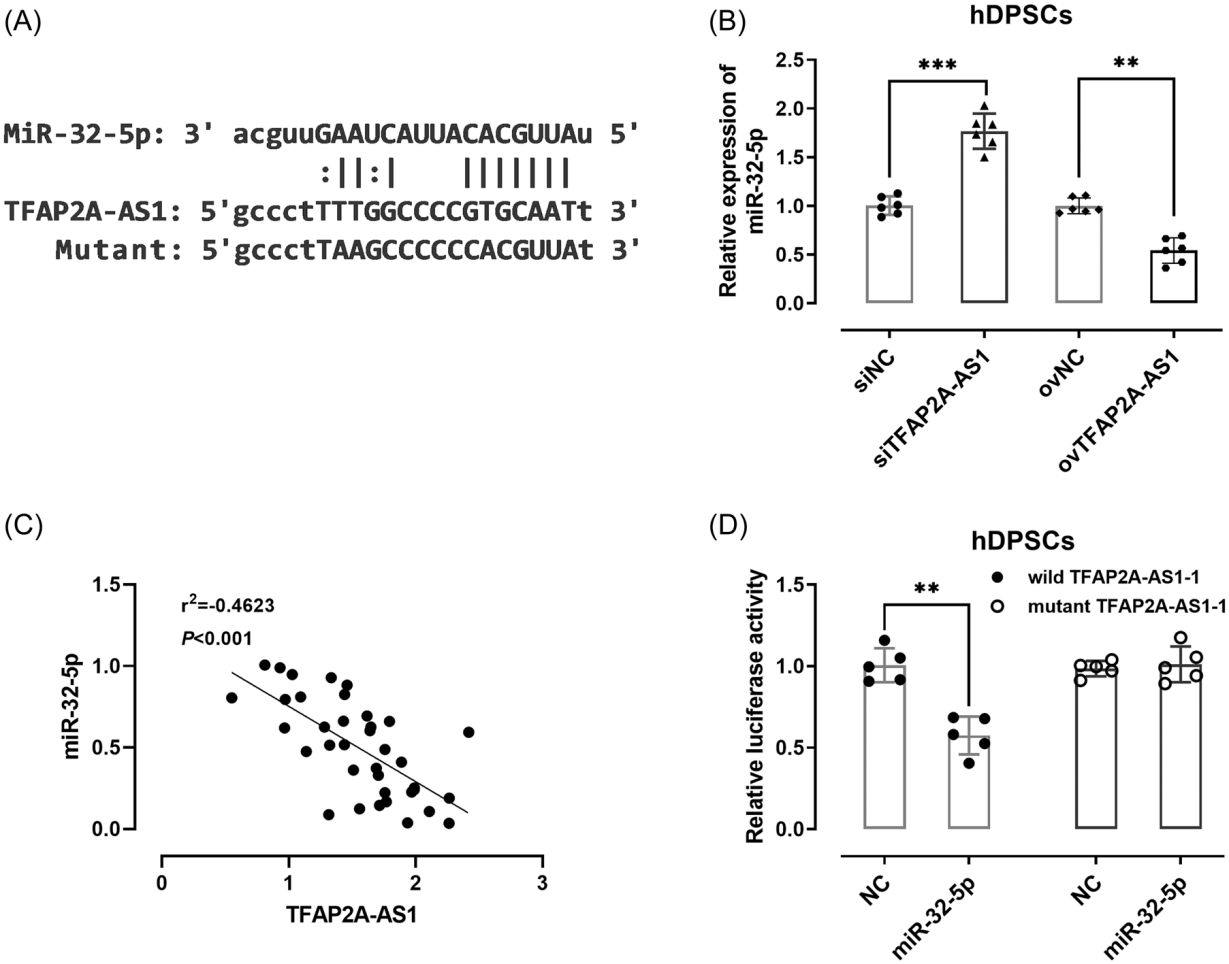
TFAP2A‐AS1 acted as a sponge for miR‐32‐5p. (A) The binding site between TFAP2A‐AS1 and miR‐32‐5p was predicted. (B) The expression level of miR‐32‐5p was analyzed using RT‐qPCR after TFAP2A‐AS1 overexpression or knockdown. (C) The expression level of TFAP2A‐AS1 was inversely correlated with that of miR‐32‐5p. (D) Relative luciferase activity was determined after cotransfection of wild or mutant TFAP2A‐AS1 with miR‐32‐5p mimics, or the corresponding control oligonucleotide in hDPSCs. **p < .01, ***p < .001. hDPSCs, human dental pulp stem cells.

Based on the search results from lncRNASNP, we focused on miR‑32‐5p (Figure 4A), which is related to inflammation^18^ and osteogenic differentiation of dental pulp stem cells.^19^ TFAP2A‐AS1 downregulation can raise the expression level of miR‐32‐5p, while its upregulation can reduce the expression level of miR‐32‐5p (*p*  <  .01) (Figure 4B). The expression level of miR‑32‐5p was inversely correlated with that of TFAP2A‐AS1 in inflamed pulp (Figure 4C). Under wild‐type TFAP2A‐AS1 condition, luciferase activity was significantly attenuated by miR‐32‐5p overexpression (Figure 4D). In contrast, no major differences of mutant TFAP2A‐AS1 were noticed regarding miR‐32‐5p‐mediated luciferase reporter gene activity.

## 4 DISCUSSION

Pulpitis is one of the most common oral diseases. Its main clinical manifestations include spontaneous and paroxysmal pain, cold‐ and hot‐stimulating pain, and nocturnal pain, which seriously affects the quality of life of patients.^20^ Previous studies prove that lncRNA is involved in the inflammatory process of the immune system, and is related to pulpitis.^21^ In a study by HUANG et al., lncRNA expression in pulpitis and normal human dental pulp tissues was assessed. They found significant differences in lncRNA expression between normal dental pulp tissue and pulpitis‐affected tissue, suggesting lncRNA could play a crucial role in the pathogenesis of pulpitis.^22^ In our study, we analyzed GSE92681 data set, and identified TFAP2A‐AS1 as a differentially expressed lncRNA in pulpitis. TFAP2A‐AS1 has been reported to be related to the immune response in breast cancer treatment and oral squamous cell carcinoma progress.^23^, ^24^ Subsequently, we identified TFAP2A‐AS1 expression profiles in pulpitis tissues and found it was upregulated in pulpitis tissues compared with the healthy pulp. After induction of odontoblastic differentiation, TFAP2A‐AS1 expression level was decreased in hDPSCs. In contrast, if hDPSCs were treated with LPS, TFAP2A‐AS1 expression level increased. These findings imply TFAP2A‐AS1 may be involved in the progress of pulpitis.

Functional pulp tissue is a prerequisite for completing root formation during tooth development and eruption. The odontoblasts lined in pulp can polarize and secrete a collagenous matrix, to physiologically and continuously form dentine.^25^ The composition of dentine is similar to that of bone. This study explored the regulatory mechanism of TFAP2A‐AS1 in the odontogenic differentiation. TFAP2A‐AS1 was overexpressed in hDPSCs, followed by differentiating into odontoblasts in vitro. The expression profiles of osteogenic markers in TFAP2A‐AS1‐downregulated hDPSCs were then examined and compared to those in non‐downregulated cells. The results showed that the upregulation of TFAP2A‐AS1 inhibited the osteo/odontogenic differentiation potential of DPSCs. Similarly, lncRNA‑Ankrd26 from dental pulp stem cells can promote dental pulp restoration through osteoblastic differentiation of mesenchymal stem cells.^26^ lncRNA SNHG1, LINC00968, and linc02349 can also promote the odontogenic differentiation of hDPSCs.^27^, ^28^, ^29^ However, other lncRNAs, such as DANCR and LINC01133, have inhibitory effects on bone differentiation of hDPSCs.^30^, ^31^ This study provides a new target for the regenerative treatment of endodontic diseases and enhances understanding of hDPSCs functions.

The cellular response to pulpal infection involves several resident cells, such as odontoblasts and fibroblasts, as well as immune cells. The recruited immune cells at the site of infection release the effector molecules including cytokines, chemokines and other pro‐inflammatory mediators.^32^ The severity of inflammation dictates the outcome of dental pulp infection, which could result in regeneration, repair, or necrosis. hDPSCs play an important role in the regenerative and inflammatory response of the pulp to trauma and injury. Increasingly studies have evaluated the role of lncRNAs in the cellular and immune mediators in diseased dental pulps.^11^ For example, an in‐vitro study revealed an upregulation of PVT1 in the pulpitis cell model, which faciliates injury to human dental pulp cells caused by LPS.^33^ This in‐vitro study demonstrated TFAP2A‐AS1 knockdown can reverse the LPS‐induced increase in inflammatory mediators. Moreover, lncRNAs may regulate the proliferation and apoptosis of hDPSCs. LncRNA H19 is known to enhance the proliferation capability of hDPSCs.^34^ In the current study, TFAP2A‐AS1 showed an inhibitory effect on proliferation capability and a promoting effect on the apoptotic capability of hDPSCs. These data provide insight into the regulatory effects of TFAP2A‐AS1 on inflammatory response and growth of hDPSCs.

Considering the ceRNA function of lncRNAs, we further investigated the potential downstream miRNA for TFAP2A‐AS1. Ultimately, miR‐32‐5p was verified to be a miRNA of TFAP2A‐AS1 in hDPSCs. In oxidized low‐density lipoprotein‐induced human umbilical vein endothelial cells, miR‐32‐5p can suppress the expression of inflammatory factors including IL‐1β, IL‐6, TNF‐α, ICAM‐1, and VCAM‐1.^18^ This indicates the anti‐inflammatory function of miR‐32‐5p in cell inflammation. Notably, miR‐32 can be activated by enamel matrix proteins and regulate osteogenic and adipogenic differentiation of human exfoliated deciduous teeth.^19^ This miRNA potentially plays an important role in hDPSCs differentiation. Therefore, it was speculated that TFAP2A‐AS1 may function in diseased dental pulps via sponging miR‐32‐5p.

The use of TFAP2A‐AS1 could serve as a promising epigenetic biomarker for diagnosing pulpitis. Moreover, its role in various biological processes of pulpitis may highlight its potential as an epigenetic therapy. However, this study, conducted in our single center, has some limitations, including a small sample size and in vitro experiments. Therefore, future research is needed to explore the roles of TFAP2A‐AS1 in pulpitis in vivo and the feasibility of a TFAP2A‐AS1‐inhibiting therapy.

## 5 CONCLUSION

This study demonstrates TFAP2A‐AS1 expression and potential function in human dental pulp. The expression pattern of TFAP2A‐AS1 in human inflamed pulp was higher than that in healthy pulp. TFAP2A‐AS1 appears to hinder odontogenic differentiation, but it promotes inflammation in pulp cells. These findings suggest that TFAP2A‐AS1 may contribute to the pathogenesis of pulpitis Further research is needed to elucidate mechanism of TFAP2A‐AS1 in pulpal diseases.

## AUTHOR CONTRIBUTIONS

**Mingming Liu**: Conceptualization; data curation; writing—original draft; writing—review and editing. **Weijing Jia**: Conceptualization; Data curation; investigation; writing—review and editing. **Lin Bai**: Conceptualization; data curation; investigation; writing—review and editing. **Qiaolin Lin**: Conceptualization; data curation; writing—original draft; writing—review and editing.

## CONFLICT OF INTEREST STATEMENT

There is no conflict of interest in this study.

## ETHICS STATEMENT

The study protocol was approved by The Ethics Committee of Shijiazhuang Fourth Hospital and followed the principles outlined in the Declaration of Helsinki. In addition, informed consent has been obtained from the participants involved.

## Data Availability

The datasets used and/or analysed during the current study are available from the corresponding author on reasonable request.

## References

[1] Thesleff I . From understanding tooth development to bioengineering of teeth. Eur J Oral Sci. 2018;126(suppl 1):67‐71. 30178557 10.1111/eos.12421

[2] Van Hassel HJ . Reprint of: physiology of the human dental pulp. J Endod. 2021;47(5):690‐695. 33892898 10.1016/j.joen.2021.03.001

[3] Zhang W , Yelick PC . Tooth repair and regeneration: potential of dental stem cells. Trends Mol Med. 2021;27(5):501‐511. 33781688 10.1016/j.molmed.2021.02.005 PMC9907435

[4] Galler KM , Weber M , Korkmaz Y , Widbiller M , Feuerer M . Inflammatory response mechanisms of the dentine‐pulp complex and the periapical tissues. Int J Mol Sci. 2021;22(3):1480. 33540711 10.3390/ijms22031480 PMC7867227

[5] Klein C , Meller C , Schäfer E . Human primary odontoblast‐like cell cultures‐a focused review regarding cell characterization. J Clin Med. 2022;11(18):5296. 36142943 10.3390/jcm11185296 PMC9501234

[6] Yoshida S , Tomokiyo A , Hasegawa D , Hamano S , Sugii H , Maeda H . Insight into the role of dental pulp stem cells in regenerative therapy. Biology. 2020;9(7):160. 32659896 10.3390/biology9070160 PMC7407391

[7] Shi X , Mao J , Liu Y . Pulp stem cells derived from human permanent and deciduous teeth: biological characteristics and therapeutic applications. Stem Cells Transl Med. 2020;9(4):445‐464. 31943813 10.1002/sctm.19-0398 PMC7103623

[8] Duncan HF , Cooper PR . Pulp innate immune defense: translational opportunities. J Endod. 2020;46(9s):S10‐S18. 32950180 10.1016/j.joen.2020.06.019

[9] Hui T , Wang C , Chen D , Zheng L , Huang D , Ye L . Epigenetic regulation in dental pulp inflammation. Oral Dis. 2017;23(1):22‐28. 26901577 10.1111/odi.12464 PMC4993683

[10] Liu Y , Gan L , Cui DX , et al. Epigenetic regulation of dental pulp stem cells and its potential in regenerative endodontics. World J Stem Cells. 2021;13(11):1647‐1666. 34909116 10.4252/wjsc.v13.i11.1647 PMC8641018

[11] Galicia J , Khan AA . Non‐coding RNAs in endodontic disease. Semin Cell Dev Biol. 2022;124:82‐84. 34257038 10.1016/j.semcdb.2021.07.006

[12] Deng P , Chen QM , Hong C , Wang CY . Histone methyltransferases and demethylases: regulators in balancing osteogenic and adipogenic differentiation of mesenchymal stem cells. Int J Oral Sci. 2015;7(4):197‐204. 26674421 10.1038/ijos.2015.41 PMC5153596

[13] Zhang P , Wu W , Chen Q , Chen M . Non‐coding RNAs and their integrated networks. J Integr Bioinform. 2019;16(3):20190027. 31301674 10.1515/jib-2019-0027 PMC6798851

[14] Sehic A , Tulek A , Khuu C , Nirvani M , Sand LP , Utheim TP . Regulatory roles of microRNAs in human dental tissues. Gene. 2017;596:9‐18. 27725267 10.1016/j.gene.2016.10.009

[15] Kearney M , Cooper PR , Smith AJ , Duncan HF . Epigenetic approaches to the treatment of dental pulp inflammation and repair: opportunities and obstacles. Front Genet. 2018;9:311. 30131827 10.3389/fgene.2018.00311 PMC6090030

[16] Liu M , Chen L , Wu J , Lin Z , Huang S . Long noncoding RNA MEG3 expressed in human dental pulp regulates LPS‐Induced inflammation and odontogenic differentiation in pulpitis. Exp Cell Res. 2021;400(2):112495. 33524362 10.1016/j.yexcr.2021.112495

[17] Dai Y , Xuan G , Yin M . DUXAP8 promotes LPS‐Induced cell injury in pulpitis by regulating miR‐18b‐5p/HIF3A. Int Dent J. 2023;73(5):636‐644. 36522211 10.1016/j.identj.2022.11.011 PMC10509439

[18] Zhang P , Luo J , Wu T , et al. MiR‐32‐5p/AIDA mediates OxLDL‐induced endothelial injury and inflammation. Int Heart J. 2022;63(5):928‐938. 36184552 10.1536/ihj.22-067

[19] Li XY , Zheng L , Ma PT , Zhang YX . Effect of enamel matrix protein on osteogenic and adipogenic differentiation of dental pulp stem cells of deciduous teeth through miR‐32. Shanghai Kou Qiang Yi Xue. 2021;30(4):367‐373. 34693428

[20] Hellyer P . Pulpotomy and pulpitis. Br Dent J. 2022;232(7):459. 10.1038/s41415-022-4164-3 35396428

[21] Zhang K , Qiu W , Wu B , Fang F . Long non‑coding RNAs are novel players in oral inflammatory disorders, potentially premalignant oral epithelial lesions and oral squamous cell carcinoma (Review). Int J Mol Med. 2020;46(2):535‐545. 32626947 10.3892/ijmm.2020.4628 PMC7307862

[22] Huang X , Chen K . Differential expression of long noncoding RNAs in normal and inflamed human dental pulp. J Endod. 2018;44(1):62‐72. 29079059 10.1016/j.joen.2017.08.022

[23] Cao J , Liang Y , Gu JJ , Huang Y , Wang B . Construction of prognostic signature of breast cancer based on N7‐Methylguanosine‐Related LncRNAs and prediction of immune response. Front Genet. 2022;13:991162. 36353118 10.3389/fgene.2022.991162 PMC9639662

[24] Jie G , Peng S , Cui Z , He C , Feng X , Yang K . Long non‐coding RNA TFAP2A‐AS1 plays an important role in oral squamous cell carcinoma: research includes bioinformatics analysis and experiments. BMC Oral Health. 2022;22(1):160. 35524329 10.1186/s12903-022-02203-4 PMC9074241

[25] Xie Z , Shen Z , Zhan P , et al. Functional dental pulp regeneration: basic research and clinical translation. Int J Mol Sci. 2021;22(16):8991. 34445703 10.3390/ijms22168991 PMC8396610

[26] Li L , Ge J . Exosome‑derived lncRNA‑Ankrd26 promotes dental pulp restoration by regulating miR‑150‑TLR4 signaling. Mol Med Rep. 2022;25(5):152. 35244185 10.3892/mmr.2022.12668

[27] Fu T , Liu Y , Huang X , Guo Y , Shen J , Shen H . lncRNA SNHG1 regulates odontogenic differentiation of human dental pulp stem cells via miR‐328‐3p/Wnt/β‐catenin pathway. Stem Cell Res Ther. 2022;13(1):311. 35841022 10.1186/s13287-022-02979-w PMC9284872

[28] Cao L , Liu W , Zhong Y , et al. Linc02349 promotes osteogenesis of human umbilical cord‐derived stem cells by acting as a competing endogenous RNA for miR‐25‐3p and miR‐33b‐5p. Cell Proliferation. 2020;53(5):e12814. 32346990 10.1111/cpr.12814 PMC7260076

[29] Liao C , Zhou Y , Li M , Xia Y , Peng W . LINC00968 promotes osteogenic differentiation in vitro and bone formation in vivo via regulation of miR‐3658/RUNX2. Differentiation. 2020;116:1‐8. 33065511 10.1016/j.diff.2020.09.005

[30] Shi Q , Zheng M . Role of LINC01133 in osteogenic differentiation of dental pulp stem cells by targeting miR‐199b‐5p. Oral Health Prev Dent. 2022;20(1):173‐184. 35481341 10.3290/j.ohpd.b2960495 PMC11641068

[31] Chen L , Song Z , Huang S , et al. lncRNA DANCR suppresses odontoblast‐like differentiation of human dental pulp cells by inhibiting wnt/β‐catenin pathway. Cell Tissue Res. 2016;364(2):309‐318. 26646542 10.1007/s00441-015-2333-2

[32] Zaky SH , Shehabeldin M , Ray H , Sfeir C . The role of inflammation modulation in dental pulp regeneration. Eur Cell Mater. 2021;41:184‐193. 33583014 10.22203/eCM.v041a13

[33] Xia L , Wang J , Qi Y , Fei Y , Wang D . Long non‐coding RNA PVT1 is involved in the pathological mechanism of pulpitis by regulating miR‐128‐3p. Oral Health Prev Dent. 2022;20(1):263‐270. 35723715 10.3290/j.ohpd.b3147193 PMC11641274

[34] Du Z , Shi X , Guan A . lncRNA H19 facilitates the proliferation and differentiation of human dental pulp stem cells via EZH2‐dependent LATS1 methylation. Molecular Therapy ‐ Nucleic Acids. 2021;25:116‐126. 34401209 10.1016/j.omtn.2021.04.017 PMC8339349

